# Perceptions of adolescents on the COVID-19 pandemic and returning to school: qualitative questionnaire survey, September 2020, England

**DOI:** 10.1186/s12887-022-03420-0

**Published:** 2022-07-29

**Authors:** Annabel A. Powell, Georgina Ireland, Felicity Aiano, Jessica Flood, Zahin Amin-Chowdhury, Joanne Beckmann, Joanna Garstang, Ifeanyichukwu Okike, Shazaad Ahmad, Mary E. Ramsay, Shamez N. Ladhani, Frances Baawuah

**Affiliations:** 1 Immunisation and Vaccine Preventable Diseases Division, UK Health Security Agency, Colindale, London, NW9 5EQ UK; 2 Specialist Children & Young People’s Services, East London NHS Foundation Trust, London, UK; 3 Birmingham Community Healthcare NHS Trust, Birmingham, UK; 4 Derbyshire Children’s Hospital, Derby, UK; 5 Manchester University NHS Foundation Trust, Manchester, UK; 6 Paediatric Infectious Diseases Research Group, St. George’s University of London, London, UK

**Keywords:** COVID-19, SARS-CoV-2, Adolescents, Pandemic perception, Testing acceptability, Infection control measures

## Abstract

**Background:**

Little is known about the views of adolescents returning to secondary school during the current COVID-19 pandemic.

**Methods:**

In September 2020, the UK Health Security Agency (UKHSA), formerly known as Public Health England (PHE),recruited staff and students in secondary schools to provide nasal swabs, oral fluid and blood samples for SARS-CoV-2 infection and antibody testing. Students aged 11–18 years in five London schools completed a short questionnaire about their perception of the pandemic, returning to school, risk to themselves and to others and infection control measures, and participating in school testing.

**Results:**

A questionnaire was completed by 64% (297/462) of participants. Students were generally not anxious at all (19.7%; 58/294) or not really anxious (40.0%; 114/295) about returning to school, although 5.4% (*n* = 16/295) were extremely nervous. Most students were very worried about transmitting the virus to their family (60.2%; 177/294) rather than to other students (22.0%; 65/296) or school staff (19.3%; 57/296), or catching the infection themselves (12.5%; 37/296). Students were more likely to maintain physical distancing in the presence of school staff (84.6%; 247/292) and in public places (79.5%; 233/293) but not when with other students (46.8%; 137/293) or friends (40.8%; 120/294). A greater proportion of younger students (school years 7–9; 11–14-year-olds) reported not being anxious at all than older students (school years 12–13; 16–18-year-olds) (47/174 [27.0%] vs 3/63 [4.8%]; *p* = 0.001). Younger students were also less likely to adhere to physical distancing measures and wear face masks. Most students reported positive experiences with SARS-CoV-2 testing in schools, with 92.3% (262/284) agreeing to have another blood test in future visits.

**Conclusions:**

Younger students in secondary schools were less concerned about catching and transmitting SARS-CoV-2 and were less likely to adhere to protective measures. Greater awareness of the potential risks of SARS-CoV-2 transmission between secondary school students potentially leading to increased risk of infection in their teachers and their household members may increase adherence to infection control measures within and outside schools.

**Supplementary Information:**

The online version contains supplementary material available at 10.1186/s12887-022-03420-0.

## Introduction

In the current COVID-19 pandemic, much of the attention has focussed on older adults who have been disproportionately affected by SARS-CoV-2, with high rates of severe disease and deaths within this vulnerable group [1–3]. Children and adolescents have accounted for less than 5% of COVID-19 cases and very few hospitalisations or deaths, even in those with underlying comorbidities [4]. Early in the pandemic, however, the role of children in infection and transmission of SARS-CoV-2 was uncertain and, therefore, most countries closed their educational settings as part of their national lockdown measures to control the spread of the virus [5, 6]. In addition to affecting their learning and education, school closures have indirect consequences on the health and wellbeing of children, affecting their social, emotional and mental health [7], which is more likely to have a bigger impact on the most disadvantaged and vulnerable families [5]. School closures have also raised safeguarding concerns, with large reductions in referrals for cases of abuse and neglect that would likely have otherwise been picked up by school staff [8].

In England, SARS-CoV-2 first emerged in late January 2020 and cases rose rapidly from early March 2020, resulting in school closures from 20 March 2020 followed by national lockdown from 23 March 2020 [9]. Cases continued to increase until mid-April 2020 and then plateaued and declined until the end of May 2020 [10]. From 01 June 2020, certain primary school years were allowed back to school followed by some secondary school years from 15 June 2020. Strict infection control practices and social distancing measures, including smaller class sizes organised into distinct bubbles limited to 15 children, were implemented to limit the risk of transmission within educational settings [10, 11]. This was successful and led to a wider re-opening of all educational settings in England in September 2020.

Unlike for primary schools, there were concerns about reopening secondary schools due to a higher risk of infection and clinical disease in adolescents compared to younger children [5, 6]. Additionally, adolescents have increased contact patterns and behaviours and are, therefore, more likely to spread the virus than younger children. Secondary schools are also substantially larger than primary schools and, therefore, have the potential for large outbreaks [12, 13]. In England, the UK Health Security Agency (UKHSA) has been conducting COVID-19 surveillance in schools since they re-opened in June 2020 (sKIDs) [14], with surveillance extending to secondary pupils (sKIDsPLUS) since September 2020 [15]. UKHSA recruited 20 secondary schools across five English regions to study infection and transmission of SARS-CoV-2 in educational settings [15]. Staff and families of students in participating schools were invited to take part by completing online questionnaires and providing nasal swabs and blood samples at recruitment and at the end of each school term. We used the opportunity of the first school visit in September 2020 to ask secondary school students in London to complete a short, anonymised questionnaire before and after they completed their tests. The questionnaire aimed to understand the students’ perception and concerns about returning to school, catching and transmitting SARS-CoV-2, adherence to recommended infection control measures and their experience of taking part in the school surveillance conducted by UKHSA.

## Methods

### sKIDsPLUS surveillance recruitment

In September 2020, UKHSA extended its school surveillance from primary schools (sKIDs) to secondary schools (COVID-19 Surveillance of Children attending secondary schools, sKIDsPLUS) [15]. Twenty secondary schools and colleges in five areas where a paediatric investigation team could be assembled (East London, North and West London, Derby, Birmingham and Manchester) were invited to take part in sKIDsPLUS. Participating schools and colleges emailed their staff, parents of secondary school children aged < 16 years, and students aged 16–18 years with a link providing information about the surveillance, along with an online consent form and recruitment questionnaire. sKIDsPLUS investigators in each region arranged a date to attend the school or college during the first two weeks of the term to collect a nasal swab, oral fluid and blood samples from all consenting staff and students. The surveillance involved three additional visits at the end of each academic term.

### Questionnaire

Students from four secondary schools and one college in North London, were asked to complete an additional short paper questionnaire (Supplement 1) whilst waiting for their investigations in September 2020. The questionnaire sought information about student concerns about catching and transmitting COVID-19, social distancing and mask wearing behaviour and testing acceptability. sKIDsPLUS investigators took a lower nasal swab and blood sample from the students and provided instructions and observed the students taking their own oral fluid samples. Local anaesthetic cream was offered to all students to numb the skin prior to blood sampling. After the investigations, the students completed the rest of the questionnaire, detailing their experience with each of the tests performed.

## Data analysis

Data from the questionnaires was data entered into Microsoft Access and analysed in Stata v.15 (Statacorp, Tx). Data are mainly descriptive and categorical. Proportions were compared using Fisher’s exact test. A multivariate logistic regression, which included reported nervousness before testing, sex and grouped school year, was used to test for predictors of pain experienced during testing. Pain was grouped into painful (“painful”) and not painful (“Uncomfortable (but not painful)” and “No discomfort / it was fine”). A *p*-value < 0.05 was considered statistically significant and was not adjusted for multiple comparisons. Unless otherwise specified, reported percentages do not include missing values (see supplement 2 for variable completeness) and denominators relate to number of respondents. Questions that are multiple choice have been stated in results. For analysis students were grouped into school years 7–9 (11–14-year-olds), school year 10–11 (14–16-year-olds) and school year 12–13 (age 16–18-year-olds).

## Results

In the five North London secondary schools, 64.3% (297/462) of students recruited into sKIDsPLUS completed a questionnaire. Survey uptake was higher in males than females (145/186 [80.0%] vs 150/273 [54.9%]; *p* < 0.001) and varied by year group (Table 1). There were fewer participants in school year 11 compared to younger secondary school years, and among years 12 – 13 in secondary schools compared to colleges.

**Table 1:** Demographics of participating secondary school students

		Tested	Respondents	Uptake (%)	Percentage of respondents
Total		462	297	64.3	100
Sex	Male	186	145	80.0	48.8
	Female	273	150	54.9	50.5
	Other/Prefer not to say	3	2	66.7	0.7
School year	Year 7	87	50	57.5	16.8
	Year 8	88	53	60.2	17.9
	Year 9	87	73	83.9	24.6
	Year 10	82	46	56.1	15.5
	Year 11	18	12	66.7	4.0
	Year 12–13 in school	19	5	26.3	1.7
	Year 12–13 in college	81	58	71.6	19.5

### Anxiety

More than half the students (60.0%; 174/295) were not anxious, with 20.0% (59/295) not at all anxious and 40.0% (115/295) not really anxious about returning to school. 35.6% (105/295) were a little anxious and 5.4% (16/295) were extremely anxious. There were no significant differences in reported anxiety by sex (*p* = 0.17) but a greater proportion of younger students (school years 7–9) reported being not at all anxious compared to students in school years 12–13(27.0%; 47/174 vs 4.8%; 3/63; *p* = 0.001) (Table 2).

**Table 2:** Anxiety around returning to school by grouped school year

	**School years 7–9**	**School years 10–11**	**School years 12–13**	**Total**
Extremely anxious	7 (4.0%)	5 (8.6%)	4 (6.4%)	16 (5.4%)
A little anxious	58 (33.3%)	26 (44.8%)	21 (33.3%)	105 (35.6%)
Not really anxious	62 (35.6%)	18 (31.0%)	35 (55.6%)	115 (40.0%)
Not at all anxious	47 (27.0%)	9 (15.5%)	3 (4.8%)	59 (20.0%)
Total	174 (100%)	58 (100%)	63 (100%)	295 (100%)

**Fig. 1:**
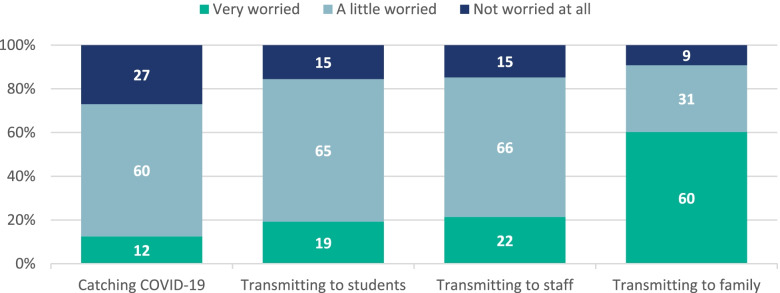
Proportion of respondents who reported being worried about catching COVID-19 and transmitting it to students, staff and family members Please note, some of the percentages may not add up to 100% due to rounding

When asked to explain their main concerns about COVID-19, most students were very worried about transmitting the virus to their family members (60.2%; 177/294) (Fig. 1).

The students were less concerned about transmitting the virus to other secondary school students (22.0%; 65/296; *p* < 0.001) or to the school staff (19.3%; 57/296; *p* < 0.001) and least concerned about catching the infection themselves (12.5%; 37/296; *p* < 0.001) (Fig. 1). There was no difference observed between grouped school years and worry about transmitting to family (*p* = 0.37), A greater proportion of students in school years 7–9 than in school years 12–13, however, reported not being worried at all about transmitting to staff (21.1%; 37/175 vs 3.17%; 2/63; *p* = 0.003) or other students (17.7%; 31/175 vs 3.2%; 2/63; *p* = 0.010), respectively. A higher proportion of female respondents reported being very worried about being infected with SARS-CoV-2 than male respondents (18.0%; 27/150 vs 6.9%; 10/145; *p* = 0.002) but there was no difference by grouped-school year (*p* = 0.66).

### Social distancing and mask wearing

**Fig. 2:**
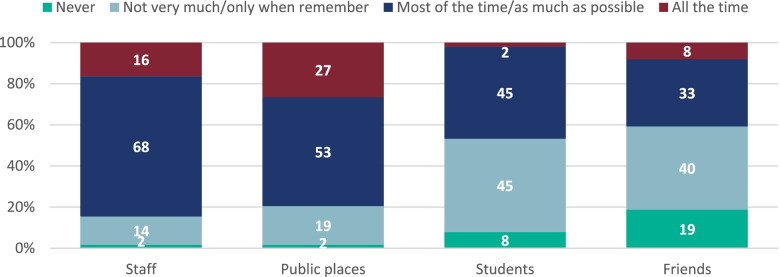
Frequency that students reported adhering to social distancing with friends, students, staff and in public places Please note, some of the percentages may not add up to 100% due to rounding

The students were asked about adherence to social distancing and wearing of face coverings and face masks in school and outside of school. Most students reported adhering to social distancing with staff (84.6%; 247/292) and in public places (79.5%; 233/293) most or all the time, but not when they were with other students (46.8%; 137/293) or with their friends (40.8%; 120/294) (Fig. 2).

However, 18.5% (55/294) of students reported never adhering to social distancing with friends. No significant differences were seen by sex however more students in school years 10–11 (20.7%; 12/58) reported never social distancing with other students than school years 7–9 (5.8%; 10/174) or students in school years 12–13 (1.6%; 1/61; *p* = 0.001).

**Fig. 3:**
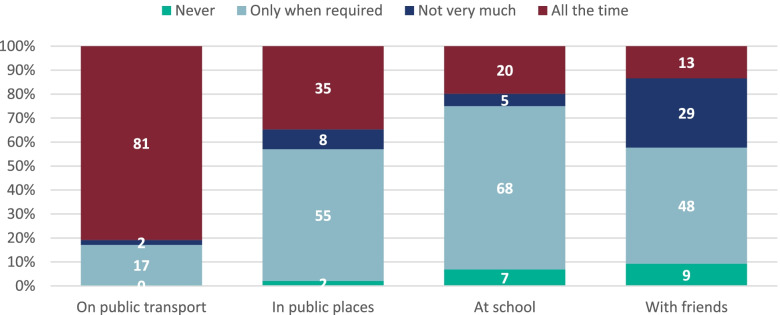
Frequency of mask use at school, with friends, in public places and on public transport Please note, some of the percentages may not add up to 100% due to rounding

All but two respondents (99.3%; 290/292) reported having a face mask. Students were able to select multiple options for types of face mask they used and most commonly reported having reusable masks (71.9%; 210/292), followed by disposable masks (34.2%; 100/292) or masks of another type (3.8%; 11/292). There were differences in patterns of mask use across different settings, ranging from 80.8% (236/292) of respondents wearing them all the time on public transport to only 13.4% (39/291) wearing them all the time with friends (Fig. 3).

A significantly higher proportion of respondents in school years 12–13 reported wearing masks all the time with friends (27.4%; 17/63 vs. 9.9%; 17/172; *p* = 0.001) and in school (30.7%; 19/62 vs. 17.9%; 31/173 *p* = 0.008) than students in school years 7–9. A higher proportion of males reported never wearing a mask in public places than females (3.6%; 5/140 vs 0.67%; 1/149 *p* = 0.003), while a higher proportion of females reported wearing a mask all the time on public transport than males (87.2%; 130/149 vs 104/141;73.8%], *p* = 0.012). In this cohort, 77.7% used one mode of transport to get to school, with 67.0% (199/297) using public transport, 45.8% (136/297) being driven or walking and 4.0% (12/297) cycling (respondents were able to select multiple options).

When asked about washing their masks, 45.2% (131/290) of students washed their masks twice a week, 34.1% (99/290) every day, 16.6% (48/290) a few times a month and 4.1% (12/290) reported never washing their mask. Mask washing was more frequently reported by females (washed every day: 60/148 [40.5%] vs 39/140 [27.9%], *p* = 0.01) but there was no significant difference by grouped school year (*p* = 0.09).

### Students’ experiences with testing in school

**Fig. 4:**
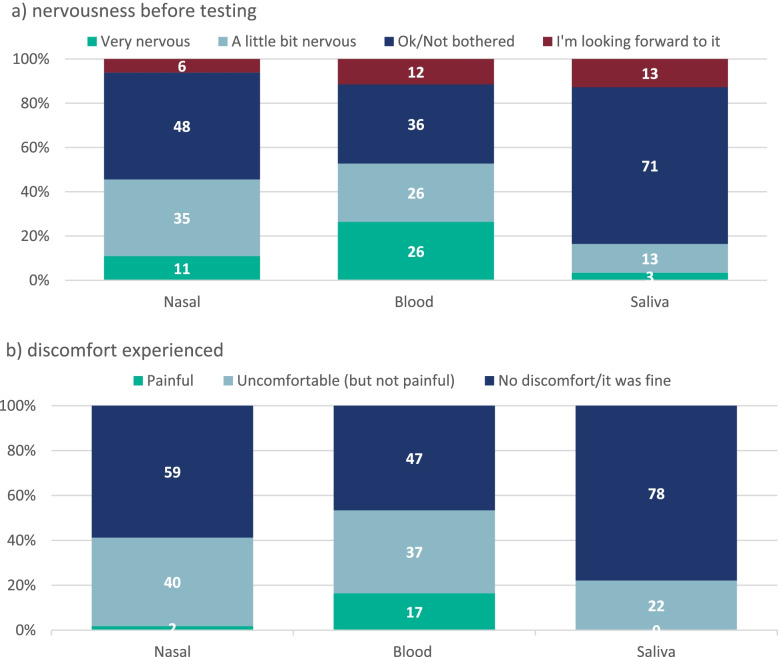
Reported nervousness about testing method before sample collection (a) and discomfort experienced during sampling Please note, some of the percentages may not add up to 100% due to rounding

Prior to having their tests, more respondents reported being very nervous about having their blood sample taken (26.4%; 78/296) than nasal swabs (10.8%; 32/294) or oral fluid samples (3.42%; 10/292) (Fig. 4).

After the test, the majority of respondents reported the tests to have caused no discomfort or being uncomfortable but not painful (Fig. 4). However, 16.5% (46/279) of respondents found having their blood taken painful. Using a multivariable logistic regression, students were more likely to report pain if they were younger and if they were nervous before taking the blood sample (Table 3).

**Table 3:** Factors associated with reported pain during blood sampling

		Adjusted^a^ Odds Ratio	95% CI
Sex	Male	1	
	Female	1.23	0.62–2.46
School year grouping	School year 7–9	1	
	School year 10–11	0.93	0.40–2.17
	School year 12–13	0.25	0.07–0.88
Nervousness before test	I’m looking forward to it	0.50	0.06–4.27
	OK/Not bothered	1	
	A little bit nervous	2.88	1.07–7.56
	Very nervous	5.59	2.08–13.4

^a^adjusted for all other factors in the table

**Fig. 5:**
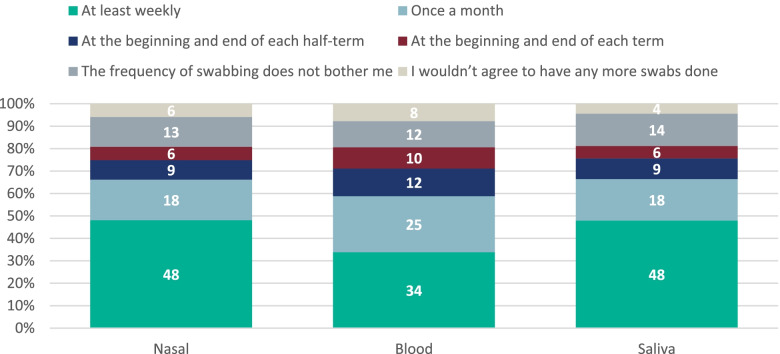
Frequency students would have blood samples, nasal swabs and saliva swabs taken Please note, some of the percentages may not add up to 100% due to rounding

When asked if they would agree to be tested again, only 7.7% (22/284) of students said they would not agree to provide blood samples again, including 40.9% (9/22) who found the blood test painful. Additionally, 5.9% (17/287) of respondents said they would not agree to nasal or and 4.4% (12/271) would not have an oral fluid test again. All other respondents reported being happy to provide samples at the scheduled study frequency or more frequently (Fig. 5).

## Discussion

Children and adolescents have been relatively spared from the direct effects of SARS-CoV-2, but there is increasing evidence emerging on the wider indirect consequences in this age group, including social isolation and mental health, which may be exacerbated by widespread school closures during the current pandemic [16]. The reopening of schools during the autumn term of 2020 was, therefore, an important step towards restoring normality in their routine lives [17, 18]. Whilst conducting detailed investigations of SARS-CoV-2 infection and transmission in educational settings, we used the opportunity to ask secondary school students participating in the sKIDsPLUS surveillance about their concerns and experiences of returning to school after a prolonged period of national lockdown in England. The high participation rates demonstrate the willingness of students and their families to help contribute to knowledge on SARS-CoV-2 infection, transmission and risks in educational settings. Others have also noted the willingness of adolescents in playing a more proactive role in the current pandemic and the importance of involving them in pandemic response [17, 19].

In this study, we found that 60% of students had some anxiety about returning to school, although the majority were only “a little anxious”. Their main concern was catching the virus and passing it to their family members. They were less worried about transmitting the virus to their friends, other students or the school staff. The older students reported being more anxious about returning to school and were more likely to maintain physical distancing and wear a face mask with friends and in school.

We also took the opportunity to assess the students’ experience with taking part in the surveillance, which included nasal swabs, oral fluid sample and blood tests, and found that the majority of them had a positive experience, especially the older students, and would continue to take part in future investigations. This was a unique situation brought about by the pandemic whereby parents consented to their child’s participation online and their children attended for SARS-CoV-2 tests in school with a member of the school staff but without their parents. The students were asked about the tests before and after the procedures and, not surprisingly, they were initially more anxious about the blood test than the nasal swab or the oral fluid sample. This was also reflected in their experience with the test itself. Those who were most anxious about the test were more likely to experience pain. Interestingly, though, of those who refused to volunteer for another blood test, more than half reported that the procedure was not painful. Overall, the acceptability of all the tests was high with only, 8% and 6% of students not willing to have another blood test and swab sample again. Moreover, a high proportion of respondents reported they would be willing to be tested more frequently than scheduled in the current investigation.

### Comparison with published literature

At the time we conducted the survey, there were limited data on the psychological and emotional impact of the pandemic on adolescents, particularly in relation to the reopening of schools after a long period of national lockdown with prolonged school closures. Reported studies include the global approach to re-opening schools after school closures [20], general effects of school closures and social isolation of children [16], the effect of the pandemic and school closures on their mental health [21, 22], and focus groups involving a small number of children [19]. We opportunistically used an ongoing school investigation to ask a number of secondary school students to complete an anonymised questionnaire which allowed us to assess multiple, albeit very focussed, issues pertaining to the reopening of schools in the current pandemic. A common emerging theme was the students’ concerns about passing the infection to others, especially those who are known to them, such as family members, but also the vulnerable in the community. In one focus group involving fifteen 11–18 year-olds, the adolescents were also concerned about social isolation, especially those with small families who relied more on their friends for social interactions as well as out-of-school activities such as sports or music classes [19]. We also found many differences in our results by gender, females were more likely in certain settings to wear masks, to wash them and a higher proportion of females reported being extremely anxious about returning to school. There is limited data on gendered anxieties around returning to school but one report does show similar trends, with school aged girls having higher anxiety scores than school aged boys [23], as well as a US study showing that school aged girls were more worried about the pandemic and about someone in their family being infected with SARS-CoV-2 than school aged boys [21]. This may make girls more likely to adhere to infection control practices such as mask wearing and washing.

The reopening of schools in England has been divisive among educationalists, parents and politicians [24]. In focus groups, adolescents appreciated the difficulties of learning from home, which were more likely to impact disadvantaged families because of limited access to technology for example, as well as the importance of face-to-face learning and the need for their parents to return to work [19]. Adolescents also understood the importance of physical distancing and infection control measures that are implemented in schools but appreciated that this was difficult to maintain outside the classrooms during school breaks and lunch times [19]. In our cohort and others, the older students acknowledged these risks more than the younger students, which may explain their increased compliance with physical distancing and mask wearing. This is consistent with a recent national survey of university students, where high compliance rates with the recommended isolation and infection control measures were reported [25]. Overall, however, adolescents continue to think that COVID-19 is not a potentially severe disease for them [26]. Additionally, it has been reported that secondary school students had a higher risk of being involved in clusters of cases or outbreaks within schools [13, 20], which highlights the need for more education and training on this topic and the importance of adhering to rules. Our results found that the students were less concerned about passing the infection to their friends, other students and teachers than to members of their family. This is important because risk perception is one of the key drivers of health behaviour and is critical for adoption of precautionary measures [27, 28]. As adolescents from our cohort were most concerned about giving COVID-19 to their families, it may prove most affective to use this key concept in health promotion messaging. Additionally, raising awareness of the low but serious risk of severe disease in adolescents, including the recently described paediatric multisystem inflammatory syndrome temporally associated with SARS-CoV-2 (PIMS-TS) [29], and consequences of severe COVID-19 in teaching staff may potentially help improve compliance to the recommended physical distancing and infection control measures among secondary school students, both within and outside the educational setting.

### Strengths and limitations

One of the main strengths is that, in comparison to other surveys which asked parents to provide information on the student’s behalf or involved small focus group, the current study involved a large number of secondary school students who completed the questionnaire themselves. In addition, the questionnaire was anonymous which reduced reporting bias, particularly for questions which about compliance with social distancing and mask wearing.

There are, however, some limitations. We only involved the North London study site for this survey, which is a multi-ethnic, multi-cultural community but it may not be representative of all secondary school students nationally and over time. Opinions may also change over time and may not reflect current views. The questionnaire was only offered to students who were taking part in the sKIDsPLUS surveillance and were, therefore, likely to be aware of their role in participating in the pandemic response. The same may also be true for participating students who completed the questionnaire compared to those who didn’t complete the questionnaire despite taking part in sKIDsPLUS. This survey was conducted at the start of the Autumn term in September when infection rates were low, since then the course of the pandemic has changed, a new variant (Alpha) was found in the UK which lead to a dramatic increase in infection rate later followed by another variants, Delta then Omicron, becoming dominant, we have had two more lockdowns and the vaccination scheme has been rolled out therefore perceptions may have changed. Finally, most questions in the questionnaire had restricted responses to allow rapid analysis of the results and may, therefore, not have allowed students to explain their views.

## Conclusions

It is important that schools remain open during the current pandemic not only to educate the students but also to support their physical, mental, emotional and social well-being. At the same time, we need to pay attention to their views and concerns on all aspects of their lives that have been impacted by the pandemic, and to provide whatever support they need during this very difficult period. By involving them, listening to them and providing them with easy access to the information they seek, adolescents can play an important part in the continued pandemic response. Their perception of risk will influence their behaviour, including compliance infection control measures to protect not only themselves but also those around them, in their households, in schools and in the wider community.

## Supplementary Information

**Additional file 1:** **Supplement 1.** Questionnaire for students. **Supplement 2.** Missingdata shown in number of respondents for each variable within the questionnaire. 

## Data Availability

The datasets generated and/or analysed during the current study are not publicly available but applications for relevant anonymised data should be submitted to the UKHSA Office for Data Release.
