# Supplementary material for: Perceptions of adolescents on the COVID-19 pandemic and returning to school: qualitative questionnaire survey, September 2020, England
Source: BMC Pediatr. 2022 Jul 29;22:456. doi: 10.1186/s12887-022-03420-0 (PMC9334543; doi:10.1186/s12887-022-03420-0)
Supplement: Supplementary file 1 — Additional file 1: Supplement 1. Questionnaire for students. Supplement 2. Missingdata shown in number of respondents for each variable within the questionnaire. [file 12887_2022_3420_MOESM1_ESM.docx]

Supplement

Supplement 1. Questionnaire for students

Skidsplus number: _________


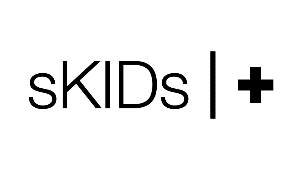


Questionnaire for Students

This is a short anonymous and confidential questionnaire.

Please answer all the questions as fully and honestly as possible.

Please complete parts 1 & 2 before your samples have been taken, and part 3 after. If you can, please do not look at part 3 until you have done all your samples.

***Part 1 – to be completed before your samples***

1. How are you feeling about the tests? *Please circle below:*

| **Nose** | **Throat** | **Blood** | **Saliva** |
| --- | --- | --- | --- |
| Very nervous  A little bit nervous  OK/Not bothered  I’m looking forward to it | Very nervous  A little bit nervous  OK/Not bothered  I’m looking forward to it | Very nervous  A little bit nervous  OK/Not bothered  I’m looking forward to it | Very nervous  A little bit nervous  OK/Not bothered  I’m looking forward to it |

1. How frequently are you prepared to do it?

| **1. How frequently would you agree to have a nose swab? (tick one)** | |
| --- | --- |
| Daily  Twice a week  Once a week  Once a month | At the beginning and end of each half-term  At the beginning and end of each term  The frequency of swabbing does not bother me  I wouldn’t agree to have any more swabs done |

| **2. How frequently would you agree to have a throat swab? (tick one)** | |
| --- | --- |
| Daily  Twice a week  Once a week  Once a month | At the beginning and end of each half-term  At the beginning and end of each term  The frequency of swabbing does not bother me  I wouldn’t agree to have any more swabs done |

| **3. How frequently would you agree to have a saliva swab? (tick one)** | |
| --- | --- |
| Daily  Twice a week  Once a week  Once a month | At the beginning and end of each half-term  At the beginning and end of each term  The frequency of swabbing does not bother me  I wouldn’t agree to have any more swabs done |

| **4. How frequently would you agree have a blood test? (tick one)** | |
| --- | --- |
| Daily  Twice a week  Once a week  Once a month | At the beginning and end of each half-term  At the beginning and end of each term  The frequency of swabbing does not bother me  I wouldn’t agree to have any more swabs done |

***Part 2 – to be completed before your samples***

1. Have you felt anxious about returning to school this term? *Please circle:*

| Extremely anxious | A little anxious | Not really anxious | Not at all anxious |
| --- | --- | --- | --- |

| 1. How do you feel about going back to school? |
| --- |
|  |
|  |
|  |
|  |

1. Are you worried about catching COVID-19?

| Not worried at all | A little bit worried | Very worried |
| --- | --- | --- |

1. How worried are you about transmitting COVID-19 to:

- other students?

| Not worried at all | A little bit worried | Very worried |
| --- | --- | --- |

- teachers/school staff?

| Not worried at all | A little bit worried | Very worried |
| --- | --- | --- |

- your family?

| Not worried at all | A little bit worried | Very worried |
| --- | --- | --- |

1. Please mark on the on the scale below how much you have been social distancing in these scenarios:

*Please answer honestly, as this is confidential, and your results are anonymous*

- At school from other students:

| All the time | Most of the time/as much as I can | Not very much/ only when I remember | Never, I don’t bother with social distancing |
| --- | --- | --- | --- |

- At school from staff:

| All the time | Most of the time/as much as I can | Not very much/ only when I remember | Never, I don’t bother with social distancing |
| --- | --- | --- | --- |

- When out in public places:

| All the time | Most of the time/as much as I can | Not very much/ only when I remember | Never, I don’t bother with social distancing |
| --- | --- | --- | --- |

- Outside school, with friends:

| All the time | Most of the time/as much as I can | Not very much/ only when I remember | Never, I don’t bother with social distancing |
| --- | --- | --- | --- |

1. Do you have a mask or face covering?

Yes No

1. If yes, what type of face mask or face covering do you have?
   - Reusable cloth
   - Disposable mask
   - Other……………
2. How often do you use your mask in these settings:

- At school

| All the time | Only when required | Not very much | Never |
| --- | --- | --- | --- |

- Outside school with friends

| All the time | Only when required | Not very much | Never |
| --- | --- | --- | --- |

- Outside school in public places

| All the time | Only when required | Not very much | Never |
| --- | --- | --- | --- |

- While using public transport

| All the time | Only when required | Not very much | Never |
| --- | --- | --- | --- |

1. How often do you wash or change your mask?

| Every day | A few times a week | A few times a month | Never |
| --- | --- | --- | --- |

1. How do you get to school? *Please tick:*
   - CarWalk
   - Bus
   - Train/tube
   - Bike
   - Other……

**Blank page – please do not continue questionnaire until you have had your samples taken – thank you! 😊**

***Part 3 - to be completed after your samples***

1. How did you find the tests? Please circle below:

| **Nose** | **Throat** | **Blood** | **Saliva** |
| --- | --- | --- | --- |
| Painful | Painful | Painful | Painful |
| Uncomfortable  (but not painful) | Uncomfortable  (but not painful) | Uncomfortable  (but not painful) | Uncomfortable  (but not painful) |
| No discomfort /  it was fine | No discomfort /  it was fine | No discomfort /  it was fine | No discomfort /  it was fine |

1. How frequently are you prepared to do the tests?

| **1. How frequently would you agree to have a nose swab? (tick one)** | |
| --- | --- |
| Daily  Twice a week  Once a week  Once a month | At the beginning and end of each half-term  At the beginning and end of each term  The frequency of swabbing does not bother me  I wouldn’t agree to have any more swabs done |

| **2. How frequently would you agree to have a throat swab? (tick one)** | |
| --- | --- |
| Daily  Twice a week  Once a week  Once a month | At the beginning and end of each half-term  At the beginning and end of each term  The frequency of swabbing does not bother me  I wouldn’t agree to have any more swabs done |

| **3. How frequently would you agree to have a saliva swab? (tick one)** | |
| --- | --- |
| Daily  Twice a week  Once a week  Once a month | At the beginning and end of each half-term  At the beginning and end of each term  The frequency of swabbing does not bother me  I wouldn’t agree to have any more swabs done |

| **4. How frequently would you agree have a blood test? (tick one)** | |
| --- | --- |
| Daily  Twice a week  Once a week  Once a month | At the beginning and end of each half-term  At the beginning and end of each term  The frequency of swabbing does not bother me  I wouldn’t agree to have any more swabs done |

| 1. Is there anything else you would like to share? |
| --- |
|  |
|  |
|  |
|  |
|  |

Thank you very much for participating in the sKIDsPLUS study. The information and samples you have given us are so important in helping us understand COVID-19 better.

**Please hand in this questionnaire to the final sKIDsPLUS table or to a member of sKIDsPLUS staff.**

Supplement 2 – Missing data shown in number of respondents for each variable within the questionnaire

|  | Missing | |
| --- | --- | --- |
| Variable | n | percent |
| Anxiety returning to school | 2 | 0.7 |
| Catching SARS-CoV-2 themselves | 1 | 0.3 |
| Transmission |  |  |
| to family | 3 | 1.0 |
| to other students | 1 | 0.4 |
| to staff | 1 | 0.4 |
| Social distancing |  |  |
| with staff | 5 | 1.7 |
| in public | 4 | 1.4 |
| with other students | 4 | 1.4 |
| with friends | 3 | 1.0 |
| Owned a face mask | 5 | 1.7 |
| Face mask use |  |  |
| on public transport | 5 | 1.7 |
| with friends | 6 | 2.0 |
| in public places | 6 | 2.0 |
| in school | 5 | 1.7 |
| Washing face mask | 7 | 2.4 |
| Feelings before sampling |  |  |
| Blood | 1 | 0.4 |
| Nasal | 3 | 1.0 |
| Oral Fluid | 5 | 1.7 |
| After sampling |  |  |
| Blood | 18 | 6.1 |
| Nasal | 16 | 5.4 |
| Oral Fluid | 56 | 18.9 |
| Frequency willing to sample (before sample was taken) | | |
| Blood | 2 | 0.7 |
| Nasal | 3 | 1.0 |
| Oral Fluid | 5 | 1.7 |
| Frequency willing to sample (after sample was taken) | | |
| Blood | 13 | 4.4 |
| Nasal | 10 | 3.4 |
| Oral Fluid | 26 | 8.8 |
